# Neurological Symptom Frequency, Cognitive Dysfunction, and Motor Impairment in Patients with Interstitial Lung Disease: A Cross-Sectional Analysis

**DOI:** 10.3390/jcm15031086

**Published:** 2026-01-30

**Authors:** Zsolt Vastag, Emanuela Tudorache, Daniel Traila, Ioana Ciortea, Ovidiu Fira-Mladinescu, Cristian Oancea, Iulia Georgiana Bogdan, Noemi Suppini, Elena Cecilia Rosca

**Affiliations:** 1 Center for Research and Innovation in Personalised Medicine of Respiratory Diseases (CRIPMRD), Pulmonology University Clinic, “Victor Babes” University of Medicine and Pharmacy, 300041 Timisoara, Romania; zsolt.vastag@umft.ro (Z.V.); ioana.ciortea@umft.ro (I.C.); mladinescu@umft.ro (O.F.-M.); oancea@umft.ro (C.O.); noemi.suppini@umft.ro (N.S.); 2 Department of Internal Medicine, Discipline of Clinical Practical Skills, “Victor Babes” University of Medicine and Pharmacy, 300041 Timisoara, Romania; 3 Doctoral School, “Victor Babes” University of Medicine and Pharmacy, 300041 Timisoara, Romania; 4 Pulmonology Department, “Victor Babes” University of Medicine and Pharmacy, 300041 Timisoara, Romania; 5 Department of Biology and Life Sciences, “Vasile Goldis” University, 310002 Arad, Romania; 6 Department of Infectious Diseases, “Victor Babes” University of Medicine and Pharmacy, 300041 Timisoara, Romania; iulia-georgiana.bogdan@umft.ro; 7 Department of Neurology, “Victor Babes” University of Medicine and Pharmacy, 300041 Timisoara, Romania; rosca.elena@umft.ro; 8 Department of Neurology, Clinical Emergency County Hospital Timisoara, 300736 Timisoara, Romania

**Keywords:** interstitial lung diseases, neurological manifestations, cognition, Montreal Cognitive Assessment

## Abstract

**Background and Objectives:** Interstitial lung diseases (ILDs) have been increasingly linked to neurological manifestations, including cognitive dysfunction and motor impairments, yet the prevalence and severity of these associations remain underexplored. We aimed to (1) compare the frequency of neurological symptoms between patients with and without ILD; (2) evaluate differences in cognitive and motor function scores; (3) perform subgroup analyses based on MoCA (Montreal Cognitive Assessment) scores; and (4) identify potential risk factors for neurological involvement. **Methods:** In this cross-sectional study, we enrolled 77 patients (40 with ILD and 37 without ILD). We recorded demographic data, smoking status, and body mass index (BMI). Neurological symptoms (tremor, diminished reflexes, paresthesia, etc.) were documented. Cognitive assessments included the MoCA and Symbol Digit Modalities Test (SDMT). Motor function was evaluated via the Berg Balance Scale (BBS), Timed Up and Go (TUG), Single-Leg Stance (SLS), and Grooved Pegboard Test (GPT). **Results:** Neurological symptoms were more prevalent in ILD (42.5%) than in non-ILD patients (16.2%; *p* = 0.003). Tremor appeared in 35% of ILD vs. 11% of non-ILD (*p* = 0.007). ILD patients showed lower mean SLS scores (7.2 ± 3.1 vs. 9.1 ± 3.8 s, *p* = 0.03) but similar TUG times (10.3 ± 2.1 vs. 9.6 ± 2.3 s, *p* = 0.20). MoCA scores < 26 were more common in those with ILDs (45% vs. 19%; *p* = 0.01). Among ILD participants, those with MoCA < 26 had significantly higher rates of tremor (51% vs. 24%, *p* = 0.04). Logistic regression revealed ILD diagnosis (OR = 3.12, 95% CI: 1.27–7.65, *p* = 0.013), older age (OR = 1.09 per year, *p* = 0.02), and smoking history (OR = 2.01, *p* = 0.05) as independent risk factors for neurological involvement. **Conclusions:** Our findings suggest that ILD is associated with a higher burden of neurological symptoms and subtle impairments in cognition and motor performance. Recognizing and addressing these manifestations may improve patient management, underscoring the importance of an integrative, multidisciplinary approach.

## 1. Introduction

Interstitial lung diseases (ILDs) encompass a heterogeneous group of diffuse parenchymal disorders characterized by varying degrees of inflammation and fibrosis, progressive dyspnea, and impaired gas exchange. Among these, idiopathic pulmonary fibrosis (IPF) represents the prototypical fibrosing ILD and is associated with substantial morbidity, mortality, and healthcare burden despite advances in diagnostic algorithms and antifibrotic therapy [1,2,3]. Contemporary guidelines and state-of-the-art reviews emphasize multidisciplinary evaluation, high-resolution computed tomography (HRCT) pattern recognition, and longitudinal monitoring of symptoms, physiology, and imaging to capture trajectories of progression across the ILD spectrum [1,2,3].

Beyond pulmonary limitation, ILDs are increasingly recognized as systemic conditions with extra-pulmonary manifestations that may meaningfully influence functional status and quality of life. Emerging work in ILD highlights neurological and neuropsychiatric features—including cognitive complaints, attentional and processing-speed deficits, and balance impairments—as clinically relevant yet understudied contributors to disability [4,5]. These observations parallel broader chronic lung disease literature and raise the possibility that neurological involvement forms part of the multisystem phenotype in fibrosing ILD.

Multiple biologic pathways plausibly link ILD to neurocognitive and motor dysfunction. Chronic and intermittent hypoxemia (from impaired diffusion, exertional desaturation, and co-existing sleep-disordered breathing) may drive cerebral hypoperfusion and white-matter injury; systemic inflammation and oxidative stress may further impair neural networks; and pulmonary vascular disease—prevalent in advanced ILD—has been associated with cognitive deficits in pulmonary hypertension cohorts [6,7,8,9]. Population-based studies also suggest that lower lung function, especially restrictive ventilatory patterns relevant to fibrotic ILD, correlates with worse cognitive performance and higher dementia risk over time [6,7].

Although early reports and scoping reviews in ILD point toward measurable cognitive impairment—even among clinically stable patients—data remain limited, heterogeneous in design, and often rely on brief screens or small samples [4,5]. For example, case–control and cross-sectional studies have described lower scores on global cognition and psychomotor measures among patients with IPF and other idiopathic interstitial pneumonias, with contributory roles proposed for hypoxemia and comorbid sleep apnea [5,9]. These preliminary findings underscore the need for standardized batteries and comparative cohorts to delineate the frequency and profile of neurological involvement in ILD versus non-ILD peers.

The choice of measurement tools is also central. The Montreal Cognitive Assessment (MoCA) is a widely used, time-efficient screen sensitive to mild cognitive impairment and executive dysfunction across clinical settings, while the Symbol Digit Modalities Test (SDMT) provides a robust index of attention and processing speed—domains frequently implicated in medical and hypoxemic populations [10,11]. Considering the motor context, the Timed Up and Go (TUG) test captures global mobility, the Berg Balance Scale (BBS) quantifies functional balance, and Single-Leg Stance (SLS) time reflects static postural control; all three are practical, validated, and informative for fall-risk profiling in respiratory populations [12,13,14]. Their combined use offers complementary insights into mobility and balance phenotypes potentially affected by ILD-related physiology.

Notwithstanding these advances, there are still important gaps: (i) few studies have concurrently profiled discrete neurological symptoms alongside standardized cognitive and motor tests in ILD; (ii) the comparative burden versus non-ILD patients recruited from the same care environment is uncertain; and (iii) risk factors for neurological involvement—including age, smoking, and body composition—require multivariable evaluation. Furthermore, respiratory-specific guidance on assessing balance and mobility in chronic lung disease highlights the clinical value of structured balance assessments in routine care, supporting their incorporation into ILD research paradigms [13,15].

It was hypothesized that adults with ILD would exhibit (a) a higher frequency of clinically relevant neurological symptoms and (b) worse performance on standardized cognitive (MoCA, SDMT) and motor/balance (TUG, BBS, and SLS) measures compared with contemporaneous non-ILD controls. The primary objective was to compare neurological symptom frequency and cognitive/motor performance between ILD and non-ILD groups; secondary objectives were to examine differences across MoCA-defined strata and to identify independent risk factors for neurological involvement using multivariable modeling.

## 2. Materials and Methods

### 2.1. Design and Ethical Considerations

This single-center, cross-sectional study was conducted at the Victor Babes University of Medicine and Pharmacy, Timisoara, and affiliated clinical services—Victor Babes Clinical Hospital for Infectious Diseases and Pneumophthisiology, Timisoara. Consecutive adults attending routine respiratory outpatient clinics and inpatient pulmonology services were screened and enrolled into two parallel groups—patients with HRCT-confirmed fibrosing interstitial lung disease and contemporaneous non-ILD comparators evaluated in the same care environment. Enrollment occurred at a single study visit, and participants were included only if clinically stable and able to complete cognitive and motor testing safely.

The protocol received approval from the Local Commission of Ethics for Scientific Research of the affiliated hospitals and complied with the Declaration of Helsinki, the EU Good Clinical Practice Directive (2005/28/EC), and International Council for Harmonisation guidelines. Written informed consent was obtained from all participants prior to any study activities. Identifiable information was stored separately from study data, and all analyses used de-identified datasets.

PICO statement: the population comprised adults evaluated at the same academic center, with or without HRCT-confirmed interstitial lung disease. The exposure was ILD diagnosis and, within ILD, the imaging phenotype was classified as UIP, NSIP, or mixed/other. The comparator consisted of contemporaneous non-ILD patients drawn from the same care pathways. The outcomes included the frequency of predefined neurological signs or symptoms, global cognition by MoCA [10], processing speed by SDMT, motor and balance performance, subgroup differences by cognitive status, and multivariable predictors of neurological involvement. The study was designed as a cross-sectional comparative analysis.

### 2.2. Inclusion and Exclusion Criteria

Eligible participants were adults aged 18 years or older who were fluent in Romanian, able to comprehend and complete neurocognitive instruments, and physically capable of safely performing the motor and balance assessments. The ILD group comprised patients with high-resolution computed tomography (HRCT) confirmation of ILD, with multidisciplinary adjudication of radiologic pattern as usual interstitial pneumonia (UIP), nonspecific interstitial pneumonia (NSIP), or mixed/other forms; only patients deemed clinically stable enough to complete testing were included. The non-ILD group included adults without clinical, physiological, or imaging evidence of diffuse parenchymal lung disease who were evaluated in the same care pathways for non-ILD conditions. Exclusion criteria at screening and on the testing day included neurological disorders likely to confound the examination or testing (such as prior large-vessel stroke with residual deficits, Parkinson’s disease, multiple sclerosis, or moderate-to-severe dementia), acute delirium or intoxication, severe visual or hearing impairment that would invalidate cognitive testing, musculoskeletal or vestibular conditions that precluded standardized balance or gait assessment, unstable cardiovascular status, current high-dose sedatives or neuroleptics that would interfere with testing validity, and refusal or withdrawal of consent.

### 2.3. Study Variables and Outcomes

The primary exposure was ILD status, defined as present or absent by HRCT and clinical adjudication; within the ILD group, the HRCT pattern (UIP, NSIP, or mixed/other) was recorded for exploratory subgroup analyses. The primary outcomes were the frequency of predefined neurological signs or symptoms, global cognitive performance measured by the Montreal Cognitive Assessment (MoCA), and information-processing speed measured by the Symbol Digit Modalities Test (SDMT) [16]. Motor and balance performance constituted co-primary functional outcomes. Secondary outcomes included subgroup differences across cognitive strata defined a priori by MoCA < 26 versus ≥26 and the strength of associations between cognitive and motor domains. Covariates captured at baseline included age, sex, body mass index (BMI), smoking status categorized as never versus current/former, and major comorbidities, alongside current medications with potential neurocognitive or motor effects such as benzodiazepines, anticholinergics, beta-blockers, and dopaminergic agents.

### 2.4. Definitions

Neurological involvement was operationalized as the presence of at least one predefined index sign or symptom identified during a brief standardized neurological examination and structured interview. Tremor was classified phenomenologically at the bedside as resting tremor (present in a fully supported limb at rest), postural tremor (present while maintaining an anti-gravity posture with arms outstretched), or kinetic/intention tremor (emerging during goal-directed movement, assessed using finger-to-nose maneuvers). Any tremor was recorded if observed under at least one of these standardized conditions. Given the clinic-based design, tremor categorization was descriptive and not intended to replace a comprehensive evaluation of movement disorders.

Cognitive testing included the MoCA, a 30-point instrument in which higher scores reflect better global cognition; a score below 26 indicated possible mild cognitive impairment and served as the predefined threshold for subgrouping. The SDMT was administered with a practice row followed by one full trial, yielding two summaries: completion time in seconds (SDMT-T, where higher values indicate slower processing) and number of correct symbol–digit matches (SDMT-C, where higher values indicate better performance).

Motor and balance testing followed standard protocols. TUG measured the time in seconds required to stand from a standard chair, walk three meters, turn, return, and sit; higher values indicated worse mobility. The BBS consisted of 14 items scored from 0 to 4, with totals ranging from 0 to 56, and higher scores reflecting better balance. The SLS recorded the best of up to three trials per leg, with participants standing with hands on their hips and eyes open for a maximum of 30 s; shorter times reflected poorer unipedal stability. The Grooved Pegboard Test [17] was administered separately for the dominant and nondominant hands, with completion time in seconds recorded for each hand after a brief practice sequence; lower times indicated better fine motor dexterity.

ILD imaging patterns were abstracted from radiology reports finalized through multidisciplinary discussion; classification into UIP, NSIP, or mixed/other was used descriptively and for exploratory within-ILD analyses. All testing took place in a quiet, well-lit room with standardized instructions and demonstrations. Examiners underwent structured training and were observed during initial enrollments to ensure protocol fidelity. Cognitive scoring sheets were concealed during administration to minimize expectancy bias, and MoCA totals were independently verified from raw responses by a second reviewer. Motor assessors were not involved in ILD adjudication. Adverse events, including loss of balance or symptomatic hypotension, were documented, and testing was paused or discontinued as needed for safety.

### 2.5. Statistical Analysis

Data were processed using SPSS (version 26) and Microsoft Excel. Continuous variables were tested for normality (Kolmogorov–Smirnov). Independent-sample *t*-tests were applied for normally distributed data; the Mann–Whitney U test was used otherwise. Categorical variables were compared via chi-square (χ^2^) tests. Post hoc analyses were performed where relevant to identify specific between-group differences. Logistic regression models evaluated risk factors for neurological involvement (presence of ≥1 significant neurological symptom). Covariates included ILD diagnosis, age, smoking (never vs. current/former), and BMI. Pearson’s or Spearman’s correlation coefficients assessed relationships between cognitive and motor variables. Statistical significance was set at *p* < 0.05 (two-tailed). Effect sizes (odds ratios [ORs], 95% confidence intervals [CIs]) are reported where applicable.

This was a clinic-based cross-sectional study with consecutive enrollment over the study period. Power was evaluated for the principal between-group comparisons using two-sided α = 0.05. With 40 ILD and 37 non-ILD participants (total n = 77), the study provides ~0.79 power to detect a moderate between-group difference in MoCA scores (Cohen’s d ≈ 0.64). For binary outcomes, the available sample provides ~0.74 power to detect the observed difference in the prevalence of ≥1 neurological symptom (42.5% in ILD vs. 16.2% in non-ILD), and ~0.74–0.77 power for the observed differences in tremor and diminished reflexes. We acknowledge that power is lower for infrequent symptoms (e.g., nystagmus), and those estimates should be interpreted cautiously as exploratory.

## 3. Results

Table 1 summarizes baseline demographic data for the ILD and non-ILD groups. While the ILD cohort was slightly older on average, the difference in age (66.2 vs. 63.5 years) did not reach statistical significance (*p* = 0.10). The male-to-female ratio remained similar in both groups, indicating no sex-based selection bias (*p* = 0.94). Furthermore, mean BMI was higher by nearly one point in the ILD group (28.5 vs. 27.6), but again, the difference was not statistically significant (*p* = 0.19). Smoking status was evaluated given its relevance as a potential risk factor for pulmonary and neurological conditions. The proportion of current smokers was modest in both groups (20% vs. 16%), and while there was a higher percentage of former smokers among ILD participants (35% vs. 27%), none of these comparisons achieved significance at *p* < 0.05.

Table 2 shows that neurological manifestations are more common overall in the ILD group compared to the non-ILD group. Tremor, in particular, was present in 35% of patients with ILD versus only 11% of those without ILD (*p* = 0.007). This difference was statistically significant and suggested a possible ILD-related pathophysiological mechanism affecting motor control or an associated autoimmune process. Likewise, diminished deep tendon reflexes were more frequent in those with ILDs (40% vs. 14%, *p* = 0.008), further implying that neuromuscular pathways might be compromised. Although paresthesias, nystagmus, blocked movements, and extrapyramidal signs were numerically higher in the ILD cohort, these did not reach the conventional alpha threshold of *p* < 0.05. The 22.5% rate of extrapyramidal signs in the ILD group could be clinically meaningful, yet *p* = 0.07 shows that it does not meet strict statistical significance in this sample.

Table 3 presents a comparison of MoCA and SDMT scores, which measure different facets of cognitive function—MoCA as a broad screening tool for mild cognitive impairment and SDMT as an index of attention, processing speed, and visual–motor integration. The MoCA scores were significantly lower in ILD patients (24.1) compared to the non-ILD group (26.2), with *p* = 0.003. A MoCA score <26 often indicates mild cognitive impairment, suggesting that nearly half of ILD patients may harbor subtle cognitive deficits. For SDMT-T, ILD participants showed significantly higher times (i.e., slower performance) compared to the non-ILD group (23.2 vs. 20.1 s, *p* = 0.02). This corroborates a decrement in psychomotor speed within ILD, potentially reflecting neuroinflammatory processes or hypoxic influences on cognitive-motor pathways. Meanwhile, SDMT accuracy (SDMT-C) showed a trend (19.5 vs. 17.8, *p* = 0.08) that did not reach the *p* < 0.05 threshold, possibly indicating that accuracy remains less affected than speed.

Table 4 outlines three principal motor function assessments. Although Timed Up and Go (TUG) averaged 10.4 s in ILD versus 9.5 s in non-ILD patients, this difference was not significant at *p* = 0.10, suggesting relatively similar global mobility. However, the Berg Balance Scale (BBS) scores were significantly higher in the ILD group (49.2 vs. 46.7, *p* = 0.04), implying slightly better or comparable functional balance task performance. This finding may initially seem counterintuitive, as one might expect worse balance in those with ILDs if neurological symptoms are more prevalent. Single-Leg Stance (SLS) was significantly shorter in ILD participants (7.2 vs. 9.1 s, *p* = 0.02), indicating they had more difficulty maintaining a unipedal stance.

Table 5 compares the Grooved Pegboard Test times for dominant (GPT-D) and nondominant (GPT-N) hands between ILD and non-ILD patients. Both results show no significant difference: ILD participants completed the task slightly faster (112 vs. 121 s dominant hand, *p* = 0.19) and (128 vs. 134 s nondominant hand, *p* = 0.33). These *p*-values suggest that, at least in this cohort, ILD was not associated with a pronounced deficit in fine motor coordination or dexterity as measured by GPT.

Table 6 displays the proportion of patients with MoCA scores below 26 (indicative of potential cognitive impairment) vs. 26 or above in each group. Among ILD patients, 45% (18/40) scored below 26, which is significantly higher (*p* = 0.01) than the 19% (7/37) in the non-ILD group. This aligns with earlier findings that mean MoCA scores are lower in those with ILDs, reinforcing the concern that nearly half of ILD patients may have mild cognitive deficits. In the non-ILD group, only 19% fell into the lower MoCA category, and 81% scored ≥26, which served as the reference for comparison. The fact that the chi-square test reached statistical significance (*p* = 0.01) underscores the magnitude of the difference in cognitive status between these groups. Further post hoc analyses revealed that ILD patients with MoCA < 26 also had a higher frequency of tremor and diminished reflexes.

Table 7 outlines selected correlations among cognitive measures (MoCA, SDMT-T), motor/balance tests (SLS, BBS, TUG, and GPT), and relevant clinical factors (age, tremor). A positive correlation indicates that higher scores on one measure align with higher scores on another (or indicates more severity if a variable is coded that way), while a negative correlation implies an inverse relationship. Notably, MoCA vs. SLS (r = +0.36, *p* = 0.004) suggests that better overall cognitive performance is associated with longer single-leg stance time. Similarly, MoCA vs. SDMT-T (r = −0.41, *p* = 0.001) indicates that lower MoCA scores correlate with slower SDMT speed (since a higher SDMT-T time means reduced speed). This reinforces the concept that cognitive impairment may parallel deficits in psychomotor performance. Furthermore, BBS correlated significantly with SLS (r = +0.44, *p* < 0.001), consistent with the notion that both tests reflect balance-related skills. GPT-D showed a modest but significant positive correlation with tremor severity (r = +0.27, *p* = 0.02), implying that more pronounced tremor may reduce dexterity performance. Lastly, age and TUG displayed a moderate correlation (r = +0.39, *p* = 0.003), consistent with the general understanding that mobility declines with advancing age.

Table 8 presents a logistic regression model examining the likelihood of displaying ≥ 1 significant neurological symptom (tremor, diminished reflexes, etc.). The model included ILD diagnosis, age, smoking status (current/former vs. never), BMI, and sex as predictors. ILD diagnosis emerged as a significant independent risk factor (OR = 3.12, *p* = 0.013), suggesting that patients with ILD are over three times more likely to have notable neurological findings compared to those without ILD, even after adjusting for age and smoking. Additionally, older age was also linked to greater neurological involvement (OR = 1.09 per year, *p* = 0.02). This aligns with the concept that aging exacerbates vulnerability to neurodegenerative or vascular pathways. Smoking history approached significance at *p* = 0.05, with an OR of 2.01, indicating that being a current or former smoker could double the risk of neurological signs compared to never smokers. Meanwhile, BMI and male sex did not significantly predict neurological symptoms, with *p* = 0.46 and 0.72, respectively.

**Figure 1:**
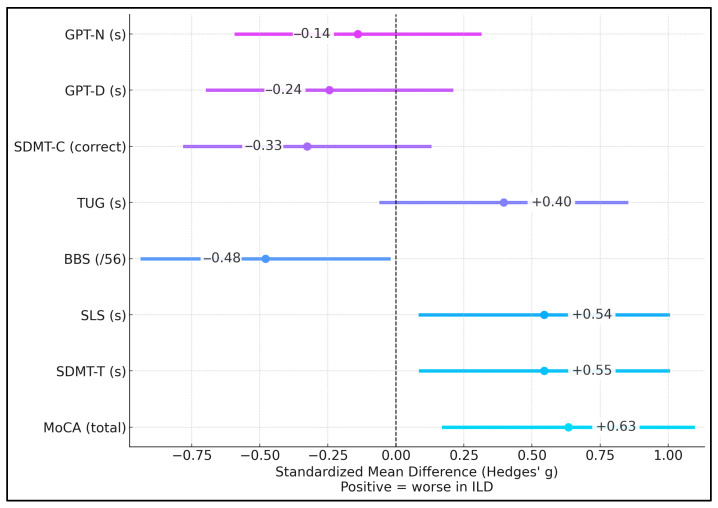
Effect-size forest plot.

It was observed that the largest standardized differences favored worse performance in the ILD group for MoCA (g = +0.61) and SDMT-T (g = +0.52), indicating lower cognition and slower processing speed, respectively. Balance SLS (g = +0.54) also showed a moderate disadvantage for those with ILDs. Mobility TUG (g = +0.39) exhibited a worse trend in ILD patients, while BBS (g = −0.49) tended to be better in the ILD group (direction harmonized, so negative implies relatively better scores for ILD on scales where higher is better). Fine dexterity differences were small, with GPT-D (g = +0.21) and GPT-N (g = +0.10), as presented in Figure 1.

Table 9 highlights how different ILD imaging patterns (UIP vs. NSIP vs. mixed/other forms) distribute within the 40 ILD patients and how frequently neurological symptoms appear in each subgroup. Usual Interstitial Pneumonia (UIP) accounted for 35% of ILD cases, while nonspecific interstitial pneumonia (NSIP) represented 45%. The remaining 20% were either mixed or unclassified patterns. Notably, 57% (8/14) of patients with UIP exhibited at least one neurological symptom, a proportion that was significant (*p* = 0.04) when compared to the other subgroups. By contrast, 33% (6/18) of NSIP patients had similar neurological manifestations (*p* = 0.08 vs. the rest of ILD), a trend but not conventionally significant. Mixed/unclassified patterns showed the lowest frequency of neurological symptoms (25%).

To examine whether ILD status was independently associated with cognitive impairment, we fitted an adjusted logistic regression model using MoCA < 26 as the outcome. After adjustment for age, sex, body mass index, and smoking history, ILD diagnosis remained associated with higher odds of MoCA < 26 (Table 10). Older age also independently contributed to MoCA < 26, while sex and body mass index showed no consistent independent association in this cohort. These findings support the interpretation that the observed cognitive signal is not explained solely by age or smoking (Table 10).

Compared with the non-ILD cohort, ILD patients showed higher symptom prevalences across domains: tremor 35.0% vs. 11.0%, diminished reflexes 40.0% vs. 14.0%, paresthesias 15.0% vs. 8.0%, nystagmus 12.5% vs. 5.4%, freezing movements 17.5% vs. 5.4%, and extrapyramidal signs 22.5% vs. 8.1%. The largest separations occurred for diminished reflexes (+26.0 pp) and tremor (+24.0 pp), reinforcing a distinct ILD-linked neurological phenotype across both pyramidal and extrapyramidal features.

## 4. Discussion

### 4.1. Analysis of Findings

The current findings—higher neurological symptom burden and worse global cognition/processing speed in those with ILDs—are concordant with emerging ILD-specific neurocognitive literature. Giannouli and colleagues showed that adults with ILD performed worse than matched healthy controls across several domains, with diffusion capacity and exercise-related oxygenation predicting cognitive performance, supporting hypoxemia and gas-exchange inefficiency as key correlates of impairment [18]. A recent scoping review synthesized six studies and highlighted factors most consistently associated with cognitive difficulties in ILD—lower DLCO, hypoxemia indices (including post-exercise PaO_2_), IPF phenotype, and sleep-disordered breathing—underscoring plausible disease-specific pathways that match our observations of more frequent MoCA < 26 in ILD [19,20,21,22,23]. A 2024 narrative review further emphasized that cognition is the most studied neurologic domain in fibrosing ILDs and that subtle deficits in attention/processing speed are common but underrecognized in routine care, reinforcing the clinical relevance of our SDMT and MoCA results [24,25,26,27].

The apparently divergent balance signals (shorter SLS in ILD despite slightly higher mean BBS) likely reflect that these tests capture overlapping but non-identical constructs. SLS is a single-task measure of static postural control that may be sensitive to subtle deficits, whereas BBS is a multi-item functional balance scale that can exhibit ceiling effects in ambulatory clinic populations and may be less sensitive to mild impairment. Interpreting these measures together therefore supports the presence of subtle balance vulnerability in ILD rather than a uniformly impaired mobility phenotype.

Motor and balance performance in our cohort showed a nuanced profile: Single-Leg Stance (SLS) times were lower in the ILD cohort (worse static balance), while TUG differences were nonsignificant, and the mean BBS score was slightly higher. This pattern aligns with test-specific properties reported in ILD; reliability work in respiratory medicine suggests that commonly used functional tests capture different constructs and have variable sensitivity to mild deficits, so complementary use is advisable rather than reliance on any single metric [20]. Clinically, SLS decrements may still matter: in general geriatric populations, inability to maintain a 10 s one-leg stance is associated with higher all-cause mortality over follow-up, suggesting that even modest SLS impairments—like those we observed—could carry prognostic or safety implications and merit targeted balance assessment/training [21].

The greater frequency of tremors and diminished reflexes in ILD patients raises mechanistic questions. While dedicated ILD studies on overt tremor are scarce, peripheral-nerve involvement is well documented across hypoxemic lung disease: COPD cohorts exhibit slowed monosynaptic reflexes and a high prevalence of peripheral neuropathy, effects linked to chronic/intermittent hypoxemia and systemic inflammation; such pathways are likely translatable to restrictive, diffusion-limited ILD physiology [22,23]. Autoimmune overlap can also contribute: in rheumatoid arthritis, neuropathy and ILD co-occur as prominent extra-articular manifestations, supporting an immune-mediated substrate for neurologic signs in some ILD subtypes [24]. A recent state-of-the-science review in fibrosing ILDs synthesizes these threads, proposing a lung–brain axis wherein hypoxemia, inflammation, and neuroimmune crosstalk jointly shape cognitive and motor symptoms—consistent with our multivariable signal for age and smoking as risk enhancers alongside ILD diagnosis [27].

An alternative, non-mutually exclusive explanation for tremor is medication effect. In respiratory populations, β-agonists can provoke or amplify fine postural/kinetic tremor, and systemic corticosteroids may contribute indirectly through sleep disruption, myopathy, or metabolic effects. We therefore interpret tremor as a clinically relevant signal that should trigger a structured review of respiratory and non-respiratory medications, caffeine intake, and thyroid status in routine care. Importantly, the co-occurrence of tremor with diminished reflexes and cognitive slowing in our cohort supports a broader neurological vulnerability rather than a single-cause mechanism.

Within-ILD analyses in our sample suggested a higher neurological symptom burden in UIP than NSIP/mixed patterns. Although direct comparative neuro-phenotyping by HRCT pattern is limited, the scoping review noted above flagged IPF as a candidate risk context for cognitive impairment, with DLCO reductions and post-exercise desaturation among the most consistent correlates [19]. These data, plus our observed SLS decrements, fit a model in which fibrotic severity and gas-exchange inefficiency mediate neurologic expression more than radiographic pattern per se—implying that physiologic staging (DLCO, exertional oximetry) may be more informative than taxonomy alone when anticipating neurologic involvement in clinic.

Correlative signals in our dataset (e.g., lower MoCA associated with slower SDMT and shorter SLS) reinforce the interdependence of cognitive and motor domains in lung disease. Contemporary perspectives advocate assessing cognition and mobility together in chronic respiratory cohorts because processing-speed/executive deficits may degrade postural control, gait safety, and self-management, even when global mobility tests like TUG remain within broad reference ranges [20]. Our regression also identified smoking history as a borderline independent risk factor. Population analyses link current smoking with poorer processing-speed performance and greater late-life cognitive risk, with cessation associated with lower incident dementia—adding a modifiable lever to address in ILD care pathways [28,29].

Clinically, our results support routine, brief neurocognitive and balance screening in ILD—MoCA and SDMT for cognition/processing speed, plus at least one static balance test (e.g., SLS) to supplement global mobility measures. Downstream, pulmonary rehabilitation remains the principal nonpharmacologic intervention with proven benefits in patients with ILDs, improving exercise capacity, dyspnea, and quality of life in systematic reviews and trials; integrating targeted balance work is reasonable when SLS or related measures are impaired [25,26]. Evidence from COPD randomized trials suggests that adding structured balance training to PR does not uniformly reduce falls, so expectations should be calibrated and programs individualized, but the signal to screen and intervene for balance limitations remains strong—particularly in patients with cognitive slowing or tremor [29].

Mechanistically, a ‘lung–brain axis’ in fibrosing ILD is biologically plausible through convergent inflammatory and hypoxic pathways. Systemic inflammation characterized by circulating cytokines (IL-6, TNF-α, and IL-1β) can alter neurovascular unit function and promote microglial activation, and chronic inflammatory states have been linked to blood–brain barrier dysfunction, increasing central vulnerability to peripheral immune signaling. In parallel, diffusion limitation and exertional desaturation can produce chronic or intermittent hypoxia that preferentially affects subcortical networks and white matter integrity, a pattern consistent with processing-speed vulnerability on measures such as the SDMT. Together, these pathways provide a coherent framework for why cognitive slowing and subtle balance deficits may cluster with neurological signs in ILD [30,31].

A further contributor may be immune dysregulation in fibrosing ILD subsets: autoimmune overlap phenotypes can plausibly affect both peripheral nerves and central neurovascular signaling, offering one explanation for heterogeneity across radiologic patterns and the higher symptom burden observed in UIP-pattern disease in our sample. Our within-ILD analysis showed a higher frequency of neurological symptoms in the UIP subgroup (57%) compared with NSIP (33%) and mixed/unclassified patterns (25%). While subgroup sizes were modest and findings should be interpreted cautiously, the pattern is clinically plausible: UIP is often associated with a more advanced fibrotic burden and progressive gas-exchange impairment, which may amplify hypoxia-related neurocognitive vulnerability. These exploratory data suggest that ILD subtype (or proxies of severity such as diffusion limitation and exertional desaturation) may help identify patients who warrant earlier, structured neurological screening.

To translate lung–brain axis concepts into testable clinical tools, future studies should pair cognitive/motor phenotyping with a biomarker panel capturing systemic inflammation, epithelial injury, neuroaxonal injury, blood–brain barrier stress, and endothelial activation. A pragmatic approach would include inflammatory markers (CRP, IL-6, and TNF-α), ILD-related epithelial injury markers (e.g., KL-6 and surfactant proteins), vascular/endothelial markers (D-dimer, VCAM-1), and neurological markers such as neurofilament light chain and S100B. Growth differentiation factor-15 (GDF-15) is particularly attractive as an integrative biomarker because it reflects cellular stress and inflammation and has been linked to cognitive risk in chronic lung disease. In ILD/IPF, circulating GDF-15 also tracks epithelial stress biology, supporting its candidacy as a cross-organ marker. Finally, extracellular vesicle and exosomal microRNA profiles (e.g., miR-21/miR-155) may provide a systems-level readout of inflammatory signaling across the lung and brain and warrant dedicated study [32,33].

Findings support integrating structured neurological screening into ILD clinics: brief tools such as MoCA and SDMT for cognition/processing speed, and SLS for balance, can identify at-risk patients. Elevated tremor and reflex abnormalities warrant targeted neurological evaluation and medication review. Older age and smoking history as risk markers argue for earlier screening and fall-risk counseling. Pulmonary rehabilitation should incorporate balance and dual-task components, while multidisciplinary pathways (respiratory, neurology, sleep medicine, rehabilitation) may improve function and quality of life.

### 4.2. Study Limitations

This single-center, cross-sectional analysis limits causal inference and generalizability. The neurological assessment was brief and symptom-focused rather than diagnostic; detailed neuroimaging, electrophysiology, and polysomnography were not performed. Potential confounders—resting/exertional hypoxemia metrics, sleep-disordered breathing severity, depression/anxiety scales, and medication timing/doses—were not fully captured. Sample sizes within ILD radiologic subtypes were small, rendering subgroup findings exploratory. Learning effects and examiner variability, though minimized via training, cannot be excluded. Subgroup analyses based on MoCA strata and HRCT patterns were prespecified but should be considered exploratory given the modest subgroup sample sizes. The neurological assessment was designed as a brief, standardized screening examination with structured symptom capture rather than a comprehensive diagnostic work-up (neuroimaging, electrophysiology, or formal evaluation of movement disorders). Accordingly, findings indicate symptom burden and screening-level signs rather than definitive neurological diagnoses.

Although several primary comparisons were adequately powered for moderate effects, the sample size limited precision for less frequent neurological signs; therefore, estimates for rare symptoms should be viewed as exploratory and require confirmation in larger cohorts. Medication exposures were recorded at the visit, but detailed dose–response and timing effects (e.g., short-acting β-agonist use immediately prior to examination) could not be fully standardized and may contribute to residual confounding. Cognitive screening is influenced by education and vascular risk burden (e.g., hypertension and dyslipidemia). Although we adjusted for major available covariates (age, sex, BMI, smoking), residual confounding by education and cardiovascular comorbidities may persist; future studies should incorporate standardized education correction and a prespecified vascular comorbidity panel. Because participants were recruited from specialized clinics and inpatient pathways, the cohort may overrepresent individuals with higher symptom burden or more advanced disease; therefore, generalizability to early or mildly symptomatic ILD should be made cautiously.

## 5. Conclusions

Adults with ILD exhibited more frequent neurological symptoms, lower global cognition, slower processing speed, and reduced static balance versus non-ILD peers, with ILD status, aging, and smoking independently linked to neurological involvement. Embedding pragmatic cognitive and balance assessments into routine ILD care is feasible and clinically informative. Future longitudinal studies should clarify mechanisms (hypoxemia, inflammation, sleep pathology) and test whether targeted interventions—smoking cessation, oxygen optimization, sleep treatment, and balance-focused rehabilitation—mitigate cognitive–motor vulnerability in ILD.

## Figures and Tables

**Table 1:** Demographic characteristics and group distribution.

Variable	ILD (n = 40)	Non-ILD (n = 37)	*p*-Value
Age, years (mean ± SD)	66.2 ± 7.1	63.5 ± 6.2	0.10 (*t*-test)
Male/Female	22/18	20/17	0.94 (χ^2^)
BMI (mean ± SD)	28.5 ± 3.3	27.6 ± 3.0	0.19 (*t*-test)
Current Smokers	20% (8/40)	16% (6/37)	0.65 (χ^2^)
Former Smokers	35% (14/40)	27% (10/37)	0.48 (χ^2^)
Never Smokers	45% (18/40)	57% (21/37)	0.31 (χ^2^)

Abbreviations: ILD, interstitial lung disease; BMI, body mass index; SD, standard deviation; χ^2^, chi-square test; *t*-test, independent-sample *t*-test.

**Table 2:** Prevalence of key neurological symptoms in ILD vs. non-ILD groups.

Neurological Symptom	ILD (n = 40)	Non-ILD (n = 37)	*p*-Value (χ^2^)
Tremor (any type)	14 (35%)	4 (11%)	0.007
Diminished Reflexes	16 (40%)	5 (14%)	0.008
Paresthesias (any limb)	6 (15%)	3 (8%)	0.31
Nystagmus	5 (12.5%)	2 (5.4%)	0.27
Blocked/Freezing Movements	7 (17.5%)	2 (5.4%)	0.1
Extrapyramidal Signs	9 (22.5%)	3 (8.1%)	0.07

Abbreviations: ILD, interstitial lung disease; χ^2^, chi-square test; *p*, two-tailed *p*-value.

**Table 3:** Cognitive test comparisons: MoCA and SDMT scores.

Parameter	ILD (n = 40; Mean ± SD)	Non-ILD (n = 37; Mean ± SD)	*p*-Value
MoCA (total)	24.1 ± 3.6	26.2 ± 2.9	0.003 (*t*-test)
SDMT-T (time)	23.2 ± 6.0	20.1 ± 5.2	0.02 (*t*-test)
SDMT-C (correct)	19.5 ± 5.4	17.8 ± 4.9	0.08 (*t*-test)

Abbreviations: MoCA, Montreal Cognitive Assessment; SDMT, Symbol Digit Modalities Test; SDMT-T, SDMT time (seconds; higher = slower); SDMT-C, SDMT correct responses (higher = better); SD, standard deviation; *t*-test, independent-sample *t*-test.

**Table 4:** Motor function tests: TUG, BBS, and SLS.

Test	ILD (n = 40; Mean ± SD)	Non-ILD (n = 37; Mean ± SD)	*p*-Value
TUG (seconds)	10.4 ± 2.2	9.5 ± 2.3	0.10 (M–W)
BBS (score/56)	49.2 ± 5.4	46.7 ± 4.9	0.04 (*t*-test)
SLS (seconds)	7.2 ± 3.1	9.1 ± 3.8	0.02 (M–W)

Abbreviations: TUG, Timed Up and Go (seconds; higher = worse); BBS, Berg Balance Scale (0–56; higher = better); SLS, Single-Leg Stance (seconds; higher = better); SD, standard deviation; M–W, Mann–Whitney U test; *t*-test, independent-sample *t*-test.

**Table 5:** Grooved Pegboard Test (GPT)—dominant and nondominant hands.

Parameter	ILD (n = 40; Mean ± SD)	Non-ILD (n = 37; Mean ± SD)	*p*-Value
GPT-D (s)	112 ± 35	121 ± 38	0.19 (*t*-test)
GPT-N (s)	128 ± 41	134 ± 44	0.33 (*t*-test)

Abbreviations: GPT, Grooved Pegboard Test; GPT-D, GPT dominant hand; GPT-N, GPT nondominant hand; s, seconds; SD, standard deviation; *t*-test, independent-sample *t*-test.

**Table 6:** Subgroup analysis by MoCA score (<26 vs. ≥26) in ILD and non-ILD groups.

Group	MoCA < 26	MoCA ≥ 26	*p*-Value (χ^2^)
ILD (n = 40)	18 (45%)	22 (55%)	0.01
Non-ILD (n = 37)	7 (19%)	30 (81%)	Reference

Abbreviations: MoCA, Montreal Cognitive Assessment; χ^2^, chi-square test; *p*, two-tailed *p*-value.

**Table 7:** Correlations (Pearson or Spearman) among cognitive and motor variables.

Pair of Variables	Correlation Coefficient (r)	*p*-Value
MoCA vs. SLS	0.36	0.004
MoCA vs. SDMT-T	−0.41	0.001
BBS vs. SLS	0.44	<0.001
GPT-D vs. Tremor (Ordinal)	0.27	0.02
Age vs. TUG	0.39	0.003

Abbreviations: MoCA, Montreal Cognitive Assessment; SDMT-T, Symbol Digit Modalities Test time (seconds; higher = slower); SLS, Single-Leg Stance; BBS, Berg Balance Scale; TUG, Timed Up and Go; GPT-D, Grooved Pegboard Test—dominant hand; r, Pearson correlation coefficient; *p*, two-tailed *p*-value.

**Table 8:** Logistic regression: predictors of neurological involvement (≥1 key symptom).

Predictor	OR (95% CI)	*p*-Value
ILD Diagnosis	3.12 (1.27–7.65)	0.013
Age (per year)	1.09 (1.01–1.18)	0.02
Smoking History	2.01 (1.00–4.03)	0.05
BMI (per unit)	1.03 (0.95–1.12)	0.46
Male Sex	1.15 (0.55–2.41)	0.72

Abbreviations: OR, odds ratio; CI, confidence interval; ILD, interstitial lung disease; BMI, body mass index.

**Table 9:** ILD imaging patterns vs. neurological symptoms.

ILD Subgroup	n (Out of 40)	UIP (%)	NSIP (%)	Other Patterns (%)	Neurological Symptoms (%)	*p*-Value (Subgroup vs. Symptoms)
UIP	14 (35%)	100%	0%	0%	57% (8/14)	0.04 (χ^2^)
NSIP	18 (45%)	0%	100%	0%	33% (6/18)	0.08 (χ^2^)
Mixed/Unclassified	8 (20%)	0%	0%	100%	25% (2/8)	0.18 (χ^2^)

Abbreviations: ILD, interstitial lung disease; UIP, usual interstitial pneumonia; NSIP, nonspecific interstitial pneumonia; χ^2^, chi-square test.

**Table 10:** Post hoc effect sizes and achieved power for key primary comparisons (n = 77).

Outcome (ILD vs. Non-ILD)	n (ILD/Non-ILD)	Observed Group Values	Effect Size (Cohen’s d or Δp)	Achieved Power (Two-Sided α = 0.05)
MoCA total score	40/37	24.1 ± 3.6 vs. 26.2 ± 2.9	d = 0.64	0.79
SDMT-T (seconds)	40/37	23.2 ± 6.0 vs. 20.1 ± 5.2	d = 0.55	0.66
Single-leg stance (seconds)	40/37	7.2 ± 3.1 vs. 9.1 ± 3.8	d = 0.55	0.66
≥1 neurological symptom (binary)	40/37	17/40 (42.5%) vs. 6/37 (16.2%)	Δp = 0.263	0.72
Tremor (binary)	40/37	14/40 (35.0%) vs. 4/37 (10.8%)	Δp = 0.242	0.72
Diminished reflexes (binary)	40/37	16/40 (40.0%) vs. 5/37 (13.5%)	Δp = 0.265	0.75

Abbreviations: ILD, interstitial lung disease; MoCA, Montreal Cognitive Assessment; SDMT-T, Symbol Digit Modalities Test.

## Data Availability

The data presented in this study are available upon request from the corresponding author.
